# Expert Performance in Action Anticipation: Visual Search Behavior in Volleyball Spiking Defense from Different Viewing Perspectives

**DOI:** 10.3390/bs14030163

**Published:** 2024-02-22

**Authors:** Ruihan Zhu, Deze Zou, Keji Wang, Chunmei Cao

**Affiliations:** 1 Department of Physical Education, Tsinghua University, Haidian District, Beijing 100084, China; zrh22@mails.tsinghua.edu.cn (R.Z.); zoudz20@tsinghua.org.cn (D.Z.); wangkeji@cnpc.com.cn (K.W.); 2 CNPC Managers Training Institute, Beijing 100096, China

**Keywords:** expert, volleyball, spike, defense, visual search behavior, eye tracking

## Abstract

Volleyball spiking requires defenders to possess exceptional anticipatory skills. However, most volleyball defense video eye-tracking studies have used fixed or off-court perspectives, failing to replicate real-world environments. This study explored different visual search behaviors between elite and novice volleyball players from various viewing perspectives using video eye tracking. We examined spiking anticipation in 14 competitive elite, 13 semi-elite, and 11 novice players. We captured spiking videos from three on-court perspectives using GoPro cameras mounted on the defenders’ heads, closely replicating real game scenarios. For comparison, we recorded baseline videos using a fixed camera. The present study revealed that competitive and semi-elite players demonstrated higher accuracy than novices. Competitive elite players used fewer fixations, indicating that their superior performance was related to stable visual search patterns. All participant groups, regardless of skill level, showed similar visual allocation among areas of interest (AOIs). However, notable differences in visual search patterns and AOI allocation were observed between baseline and on-court perspective videos. From the baseline perspective, the participants primarily utilized global perception and peripheral vision, focusing more on the setter zone or the spiker’s trunk. Conversely, from the on-court perspective, they employed more fixations, focusing more intensely on the spiker’s detailed movements.

## 1. Introduction

Volleyball is a highly competitive team sport, distinguished by its volatile and fast-paced nature, involving complex tactical interactions. Volleyball players must quickly adapt to transitions between offense and defense and execute various technical actions, including spiking, blocking, and passing. Given the inherent complexity of volleyball, athletes must be in excellent physiological condition [1] and have remarkable perceptual–cognitive skills, such as quick anticipation, under immense time pressure [2,3]. In sports-specific contexts, athletes’ perceptual–cognitive abilities, particularly their visual search skills, enable them to select and execute appropriate responses by identifying environmental cues and integrating this information with existing knowledge, thereby facilitating effective anticipation and decision making [4,5]. 

The expert–novice paradigm is frequently employed in the field of sports science to explore how prolonged training endows experts with specific characteristics resulting in superior athletic performance [6]. Behavioral studies have consistently demonstrated that athletes tend to exhibit superior perceptual–cognitive abilities, particularly in sports-specific contexts [7,8,9]. In addition to subjective methods, such as verbal reports and interviews [10], eye-tracking devices can be used as scientifically objective instruments to measure expert athletes’ visual search skills in sports-specific anticipation and decision making. Some studies have integrated both approaches for a comprehensive analysis, thereby mutually reinforcing the findings [11]. Eye-tracking technology [12] exhibits exceptional temporal resolution, enabling the precise collection of eye movement data as participants engage in visual search tasks, including fixation duration for different areas of interest (AOIs). Fixation occurs when a person’s gaze is concentrated on a single point within their field of vision for at least 100 ms [13]. The number of fixations varies depending on the task performed and the environment perceived; thus, it will increase as the task under observation becomes more challenging [14]. A long fixation duration indicates extensive visual information processing [15]. Consequently, the proportion of fixation durations among different AOIs suggests the relative importance of key visual allocation areas. Numerous eye-tracking studies have applied the temporal occlusion paradigm [16,17], which can be used to evaluate experts’ proficiency in perceiving early cues for rapid anticipation by letting participants predict the follow-up action while the video is paused at early time points [18].

Multiple sport perceptual–cognitive studies in tennis [16,19,20,21], badminton [22,23], squash [2], soccer [3,24,25], handball [26], and basketball [8] have demonstrated that expert athletes possess superior visual cognitive abilities, enabling them to effectively process early information and exhibit greater accuracy in anticipation and decision making compared to novices [5,7,27]. To explore the mechanism behind expert athletes’ exceptional anticipation performance, several studies have employed eye-tracking devices to focus on visual information utilization and processing [13,28]. Experts typically employ more efficient visual search patterns than novices in several sports anticipation tasks [3,22,29,30]. Expert athletes generally have fewer fixations and longer durations than those of novices [4,31]. However, some studies have reached different conclusions, indicating that experts’ gaze patterns may be task-dependent [27]. When eye-tracking tasks are conducted using video materials, experts tend to have fewer but longer fixations due to simpler visual cues in the video material. However, in in situ eye movement studies where the environment is more complex and more cues need to be attended to, experts typically have more, yet shorter, fixations to process the situational information more efficiently [32]. Previous studies have also shown that experts tend to direct their gaze toward task-relevant areas, thereby reducing the attention given to task-irrelevant information [28].

Research findings on volleyball expertise also demonstrate that experts generally display effective visual search patterns; however, this can vary according to forms of task presentation [11,33,34]. Advanced perceptual–cognitive skills are essential for volleyball defense, as players must rapidly recognize the opposing team’s tactics and predict the spiker’s actions to make proper defensive decisions. On the volleyball court, defensive players can be classified into two types with different responsibilities and visual search behaviors: front-row defenders (also known as blockers) and back-row defenders.

Effective blocking is a key technical skill that can directly lead to scoring points [35,36,37]. For blockers, the positional relationship between the setter and the ball serves as the key source of information for knowing the intention and tempo of the pass. A video eye-tracking study [14] found that blockers can exhibit greater accuracy in predicting the passing direction when their gaze is more focused on the ball and the setter’s wrists and less on the setter’s head. Another video study [38] showed that experts displayed fewer fixations with longer fixation durations, similar to effective visual search patterns. An in situ blocking study [39] found that experts focused more on the setter and spiker, whereas novices tended to gaze at the ball more. 

Although back-row defense might not lead to scoring directly, it significantly enhances the quality of the initial pass, indirectly boosting the success rate of the offense. Back-row defenders may have visual search strategies similar to those of blockers in the pre-spike phase. However, as the spiker hits the ball (while the blocker jumps), back-row defenders may adjust their position by shifting their attention to the spiker’s kinematics to anticipate the ball’s trajectory. When interviewed [10], top-level beach volleyball experts indicated that the situational context and opponents’ movements represented key visual information for decision making. The ball and the spiker’s shoulder, arm, elbow, wrist, and hand are all important AOIs [34]. Another video study [40] found that experts used fewer fixations with longer durations and focused more on the functional spaces between players, indicating they were adept at utilizing visual pivots and had superior peripheral vision. This conclusion was also supported by other studies [34,41]. Furthermore, as the action of serving in tennis is similar to that of spiking in volleyball, research on tennis serve reception can also provide some reference. Tennis experts allocate their attentional resources more quickly than novices [42], focusing more on the ball, the opponent’s head and shoulders, and the racket, areas that are critical for accurately predicting the ball’s trajectory [21]. 

Volleyball defense research has used two eye-tracking approaches: in situ and video. Although some studies have attempted to bridge the gap between laboratory settings and reality use in situ eye tracking [11,39,43], the complexity of real games makes it difficult to replicate the same scenarios for each participant, hindering the ability to control for the experimental variables. Video eye-tracking studies primarily utilize two types of perspectives for video materials: off-court perspectives such as the baseline [41] or sideline [14,44], and the on-court perspectives of back-row defenders or blockers. However, these studies have some limitations. First, most research has been confined to one or two perspectives, such as only Zone 1 [45] or 6 [33,34]. Second, as these studies used fixed cameras, the angles could not move with the changing contexts of the game; thus, dynamic perspectives could not be simulated. The receivers’ positioning and movement have the most influence on the efficacy of reception [46]. In addition, the visual contexts that receivers encounter differ based on their positions, potentially leading to different anticipation skills and visual search patterns, such as attention allocation among AOIs. Therefore, research from different perspectives is necessary.

The present study investigated visual search behaviors in spike defense anticipation from different back-row viewing perspectives, including one baseline off-court perspective and three on-court perspectives. The baseline perspective video was recorded using a fixed camera—similar to that used in previous studies—to act as a standard reference. To capture the authentic and dynamic on-court perspectives, we equipped the defenders in Zones 1, 6, and 5 (right back, middle back, and left back, respectively) with GoPro cameras on their heads, allowing them to make appropriate movements according to the game. This study used a differential approach, aiming to examine the disparities in anticipatory skills and visual search behaviors among competitive elites, semi-elites, and novices. It further explored the importance of different AOIs from various viewing perspectives. We hypothesized that as sports expertise increases, participants would demonstrate higher accuracy and more effective visual search strategies, especially from on-court perspectives. Furthermore, we posited that the visual fixation proportions within different AOIs would vary with changes in viewing perspectives.

## 2. Materials and Methods

### 2.1. Participants

Sample size was calculated using G*Power 3.1 [47], with an effect size set of 0.25, a desired statistical power of 0.80, and a significance level of 0.05. This analysis yielded a requisite sample size of over 30 individuals. We recruited 38 participants from Tsinghua University for this study. School coaches and club leaders were contacted, and notices were posted to recruit eligible volunteers for the experiment. Participants received monetary compensation based on their level of volleyball expertise: USD 20 for elites and USD 15 for novices. A questionnaire was used to collect each participant’s information, including training experience, performance at the highest level, and success at the highest level. According to a classification model that defined different types of elite athletes in a previous study [48], we applied an equation to quantify each participant’s expertise level based on their information. Subsequently, participants were divided into three groups based on their expertise scores: competitive elites (CE), semi-elites (SE), and novices. The CE group consisted of 14 experienced senior volleyball athletes (9 males, 5 females; mean age = 21.71 ± 2.02; expertise score = 5.46 ± 0.67) from the first volleyball team at Tsinghua University. The first team from Tsinghua University consists of athletes who were specifically recruited for their high-level professional volleyball skills, with most players advancing to join national and provincial teams upon graduation. They have won multiple championships in the national competitions, such as China University Volleyball Association. The SE group included 13 players from the second volleyball team at Tsinghua or principal members from departmental teams (10 males, 3 females; mean age = 21.54 ± 2.87; score = 3.23 ± 0.30). Their coach is a retired member of the national team; they train professionally and regularly and have won many regional and university level competitions. The novice group included 11 student enthusiasts with no formal volleyball training (8 males, 3 females; mean age = 22.82 ± 1.60; expertise score < 1). All participants had normal or corrected-to-normal vision and reported no history of neurological or psychiatric disorders. Before taking part in the study, all the participants signed a participant information sheet and an informed consent form.

### 2.2. Experimental Materials

#### 2.2.1. Experiment Equipment

Three GoPros (San Mateo, CA, USA, portable cameras with a resolution of 4K and a refresh rate of 24 Hz) and a SONY FDR-AX45 camera (Tokyo, Japan, 4K, 24 Hz) were used to capture experimental stimuli. Eye-tracking experiments were conducted using the Tobii Pro Spectrum [49] (Stockholm, Sweden, sampling rate: 1200 Hz, demonstrated in previous studies to meet the experimental requirements). 

#### 2.2.2. Video Production

The video materials were recorded at Tsinghua Indoor Volleyball Stadium. The motor experience of the spiker appears to be influential for the success rate of spiking [50]; therefore, we invited four male competitive elites from the first Tsinghua University team to be setters and spikers, as well as six male semi-elites from the second team as defensive players. Players involved in filming the video did not participate in the anticipatory eye-tracking experiment.

**Figure 1:**
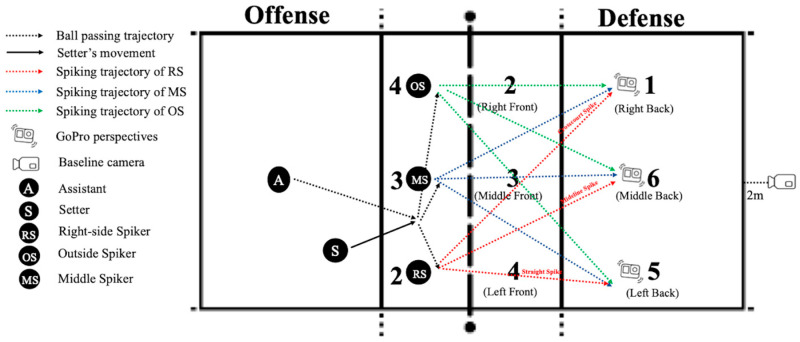
Video recording process. Notes: The figure depicts each spiking video shoot. The left side provides explanations corresponding to the figure legend.

To simulate a realistic volleyball game scenario, the offensive team consisted of one setter and three spikers, positioned as the right-side spiker, middle spiker, and outside spiker in Zones 2, 3, and 4, respectively (Figure 1). Each video recording began with the assistant passing the ball, and the setter running from the backcourt to the optimal position and passing the ball to one of the three spikers. Subsequently, the corresponding spiker executed the spike. The red, blue, and green dashed lines illustrate the diverse spike trajectory options of the three spikers. For example, the right-side spiker (red line) could choose among straight, midline, and crosscourt spikes. 

Consistent with a typical defensive arrangement in professional volleyball, the defensive team had six players: three front-court players dedicated to blocking at Zones 2, 3, and 4; and three back-court players responsible for back-row defense at Zones 1, 6, and 5. The three back-court players wore head-mounted GoPro cameras to record the videos from an on-court perspective. These cameras were affixed near the players’ eyebrows with their angles meticulously adjusted parallel to the players’ line of sight to depict their head movements and visual tracking more authentically. The defensive players were instructed to adjust their positions in response to the movements of the setter and spiker, thereby simulating real-game dynamic perspectives. A fixed camera was simultaneously set up 2 m behind the baseline of the court to record videos from a baseline perspective.

#### 2.2.3. Video Clipping

We used Apple Movie software to edit our video materials. Each video started precisely when the ball left the assistant’s hand and stopped when the spiker’s hand made contact with the ball. Each video was approximately 2.7 s in duration, with the final frame frozen for an additional 3 s. Therefore, the total duration of each video was approximately 5.7 s. The purpose of setting the final freeze-frame in this study is to compare the visual attention differences among different areas of interest (AOIs) when the offensive spiker makes contact with the ball, thereby revealing the key body kinematics information. The final freeze-frame is essential as it allows the fixation variable data sufficient time to accumulate. Considering the deviation from real game scenarios due to this freeze, we instructed participants to simulate a timely response in a real game and minimize prolonged focus on the final frame to reduce the impact of this limitation.

We recorded 35 spike actions from four different perspectives, resulting in a total of 140 videos. After meticulously reviewing the videos, we excluded 36 that did not meet our criteria, such as those with unstable passing, unclear spike trajectories, camera frame loss, and key information loss. Consequently, 104 videos were incorporated as the final stimulus materials (Table 1). We categorized the 104 videos according to spiker (Zone 2, 3, and 4), spike type (crosscourt, midline, and straight), and video perspectives (Baseline, Zone 1, 6, and 5) (Table 1).

### 2.3. Experimental Design and Process

**Figure 2:**
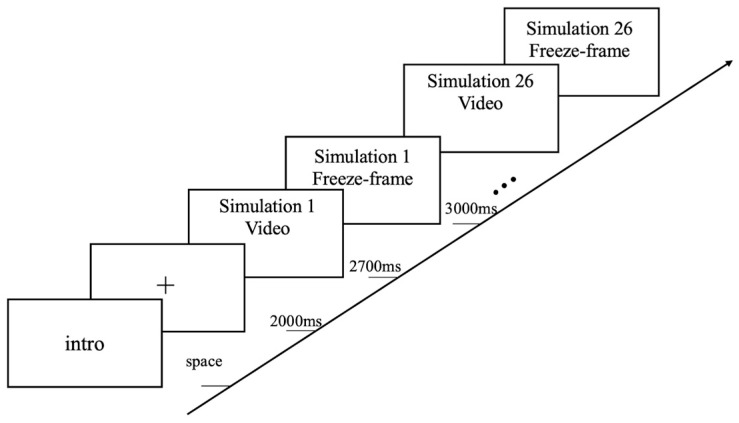
Experimental design and procedure for each section.

**Figure 3:**
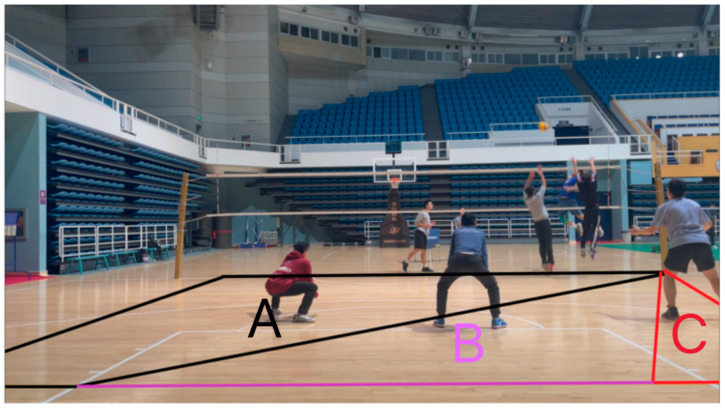
Three landing locations of the ball when the Zone 4 spiker spiked. Notes: Three areas for participants to predict where the ball would land (Key 1 = A; Key 2 = B; Key 3 = C).

The spike anticipation tasks were divided into four sections according to shooting perspectives, with the videos recorded in each section from the same perspective. The experimental procedures for each section are illustrated in Figure 2. In each section, the participants first read the introduction to the experiment and then pressed the spacebar to begin the formal experiment. Then, a focus point (“+”) would appear for 2 s before the video stimulus material was played. The participants were required to press a key (1, 2, or 3) within 5.7 s before the freeze-frame ended to predict where the ball would land (A, B, or C, respectively, as shown in Figure 3). The areas A, B, and C corresponded to the trajectory and landing location of three different spikes from the Zone 4 spiker, including crosscourt, midline, and straight spikes, respectively. Once the participants pressed a key, they would automatically see the fixation point (“+”) to start the next video. Each section included 26 videos (Table 1). The participants could rest between sections to ensure that they remained in an optimal state for the experiment.

### 2.4. Areas of Interest

**Figure 4:**
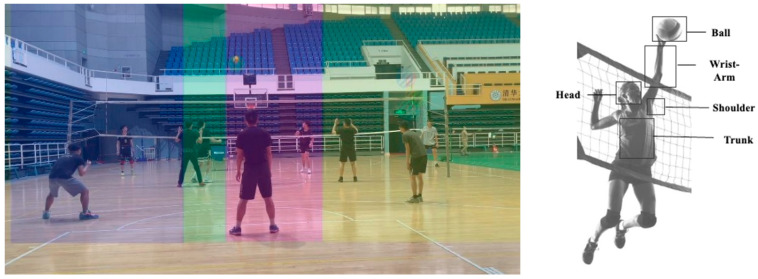
Different areas of interest. Notes: Areas of interest in the pre-spike stage (**left**). From left to right: blue, Zone 2 spiker; green, setter; red, Zone 3 spiker; yellow, Zone 4 spiker. Areas of interest in the spiking stage (**right**).

Based on a previous volleyball study [10], we divided each video into pre-spike and spiking phases for analysis (Figure 4). The pre-spike phase was defined as the period from the start of the video until the spiker contacted the ball, which lasted approximately 2.7 s. This stage had four AOIs, including the spiker in Zone 2, the setter, the spiker in Zone 3, and the spiker in Zone 4 [39,41]. The spiking phase began with the still frame and continued until the participant pressed a key. This period was used to analyze the kinematic details of the spikers on whom the participants had focused [14]. The AOIs included the ball and the spiker’s head, trunk, wrist–arm, and shoulder.

### 2.5. Statistical Analyses

Tobii Pro Lab 1.217, the analysis software for the Tobii Pro Spectrum system, was used to automatically generate the experimental data for each participant. The dependent variables in this experiment included behavioral data (i.e., response accuracy [RA] and response time [RT]), whole eye-movement data (i.e., number of fixations, total fixation duration, and first fixation duration), and fixation duration proportion across different AOIs. Fixation is defined as the concentration of an individual’s gaze on a target within their field of vision for a minimum duration of 100 ms. The number of fixations represents the quantity of distinct targets that the observer focused on. Meanwhile, the total fixation duration indicates the cumulative time spent focusing on all targets, and the first fixation duration specifies the time allocated to the initial target observed. The statistical analyses were performed using SPSS 27.0. We used the Shapiro–Wilk test and Levene’s test to assess the normality and the homoscedasticity in our data. Additionally, we used Mauchly’s test and the Greenhouse–Geisser for the sphericity correction of the sample. Our data met the statistical requirements to use the repeated measures ANOVA.

The present study was a differential study. The differential effects on the behavioral and whole eye-movement data were assessed using a two-way 3 (group) × 4 (perspective) repeated measures ANOVA and subsequent post hoc analyses. The differential effects of the group and shooting perspectives on the fixation proportion across different AOIs were assessed using a three-way 3 (group) × 4 (perspective) × 4 or 5 (AOIs) repeated measures ANOVA and subsequent Bonferroni post hoc analyses [51]. A *p*-value < 0.05 was considered statistically significant. Effect sizes were assessed using eta-squared [52] (*η*^2^ = 0.01: small effect size; *η*^2^ = 0.06: medium effect size; *η*^2^ = 0.14: large effect size). We used G*power to calculate the required effect size, with a total sample size of 38, targeting a desired statistical power of 0.80 and a significance level of 0.05. This analysis yielded a required Cohen’s *f* effect size exceeding 0.22, which, when converted to *η*^2^, corresponds to a value of 0.05.

## 3. Results

### 3.1. Behavioral Data

**Figure 5:**
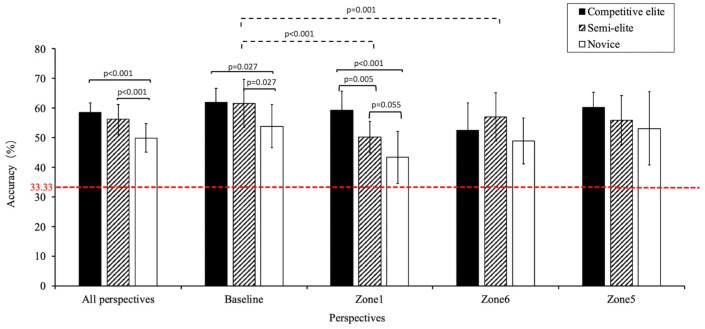
Response accuracy rates by group and perspective. Notes: The *p*-value above the solid line indicates the significance of the difference between groups; the *p*-value above the red dashed line indicates the significance of the difference between viewing perspectives.

The mean RA, regardless of groups and perspectives, was 55.2% ± 5.6%, significantly surpassing the chance level of 33.33% (Figure 5, red dashed line) (*t* = 24.200, *p* < 0.001). 

The repeated measures ANOVA with factors 3 (group) × 4 (perspective) demonstrated a notable interaction effect on RA (*F*(6,68) = 2.204, *p* = 0.050, *η*^2^ = 0.163). Significant differences were noted among the groups when the participants anticipated from the baseline (*F*(2,35) = 5.285, *p* = 0.010, *η*^2^ = 0.232) and Zone 1 (*F*(2,35) = 17.214, *p* < 0.001, *η*^2^ = 0.496). From the baseline perspective, both the CE (61.9% ± 4.7%) and SE (61.5% ± 8.1%) groups significantly outperformed the novice group (53.9% ± 7.3%). From the Zone 1 perspective, the CE group (59.3% ± 6.3%) outperformed the SE (50.3% ± 5.3%) and novice (43.4% ± 8.8%) groups (Table S1).

The main effects of group and perspective on RA were both significant (*F*(2,35) = 12.540, *p* < 0.001, *η*^2^ = 0.417); *F*(3,33) = 10.518, *p* < 0.001, *η*^2^ = 0.489). Post hoc analysis revealed that the CE (58.5% ± 3.3%) and SE (56.3% ± 5.0%) groups were significantly superior to the novice group (49.9% ± 4.8%); however, the difference between the CE and SE groups was not statistically significant. The RA from the baseline perspective (59.5% ± 7.5%) was significantly higher than that from Zones 1 (51.6% ± 9.4%) and 6 (53.0% ± 8.9%) (Table S1).

The overall mean RT was 3620.79 ms ± 198.51 ms. The ANOVA results indicated that neither the interaction effects (*F*(6,68) = 1.014, *p* = 0.424) nor the main effects (perspective, *F*(3,33) = 0.524, *p* = 0.669; group, *F*(2,35) = 1.027, *p* = 0.369) were significant.

### 3.2. Whole Fixation Data

#### 3.2.1. Total Fixation Duration of Each Video

The interaction effect with factors 3 (group) × 4 (perspective) on the total fixation duration of each video was not significant (*F*(6,68) = 1.460, *p* = 0.205), and the group main effect was also not significant (*F*(2,35) = 1.722, *p* = 0.193). However, the perspective main effect was significant (*F*(3,33) = 11.050, *p* < 0.001, *η*^2^ = 0.501). Post hoc analysis indicated that the fixation duration was longer from the baseline (2512.51 ms ± 357.66 ms) than from on-court perspectives (Zone 1: 2358.31 ms ± 379.35 ms, Zone 6: 2294.81 ms ± 425.61 ms, Zone 5, 2305.99 ms ± 434.00 ms) (Table S2).

#### 3.2.2. Number of Fixations

**Figure 6:**
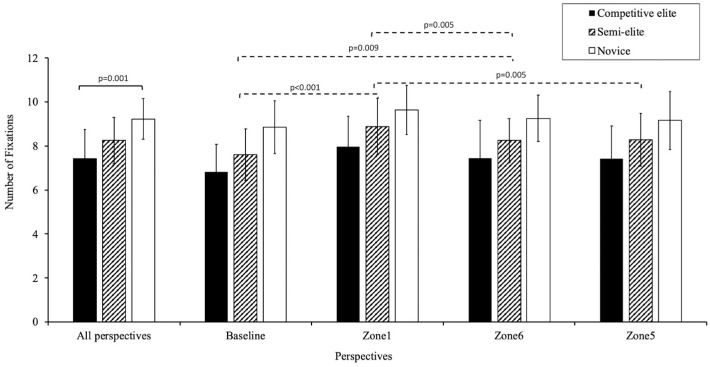
Number of fixations by group and perspective. Notes: the *p*-value above the solid line indicates the significance of the difference between groups; the *p*-value above the dashed line indicates the significance of the difference between viewing perspectives.

No significant interaction effect was revealed between groups and perspectives (*F*(6,68) = 0.317, *p* = 0.927); however, significant main effects were observed for both groups (*F*(2,35) = 7.670, *p* = 0.002, *η*^2^ = 0.305) and perspectives (*F*(3,33) = 15.403, *p* < 0.001, *η*^2^ = 0.306). The CE group (7.42 ± 1.34) exhibited significantly fewer fixations than the novice group (9.22 ± 0.93). The analysis showed the most fixations from Zone 1 (8.76 ± 1.41) and the fewest from the baseline (7.68 ± 1.43) (Figure 6, Table S3).

#### 3.2.3. First Fixation Duration

A significant interaction effect was observed between groups and perspective (*F*(6,68) = 2.391, *p* = 0.033, *η*^2^ = 0.120). A significant main effect was found for perspective (*F*(3,33) = 8.437, *p* < 0.001, *η*^2^ = 0.194) but not group (*F*(2,35) = 1.287, *p* = 0.289). Simple effect tests showed significant differences across perspectives in experts (*F*(3,33) = 10.002, *p* < 0.001, *η*^2^ = 0.476). The first fixation duration was significantly longer from the baseline perspective (296.52 ms ± 72.35 ms) than from on-court perspectives (Zone 1: 221.42 ms ± 52.16 ms, Zone 6: 242.23 ms ± 53.00 ms, Zone 5: 239.00 ms ± 55.05 ms) (Table S4).

### 3.3. Fixation Proportion of Areas of Interest

To measure the attention allocation among different AOIs, a new dependent variable of “fixation proportion” was constructed using the fixation duration as follows:

$$\mathrm{Fixation}\;\mathrm{proportion}\;(\%)=\frac{Fixation\;duration\;of\;AOI\;\left(ms\right)}{Total\;Fixation\;duration\;\left(ms\right)}$$

#### 3.3.1. Pre-Spike Phase

**Figure 7:**
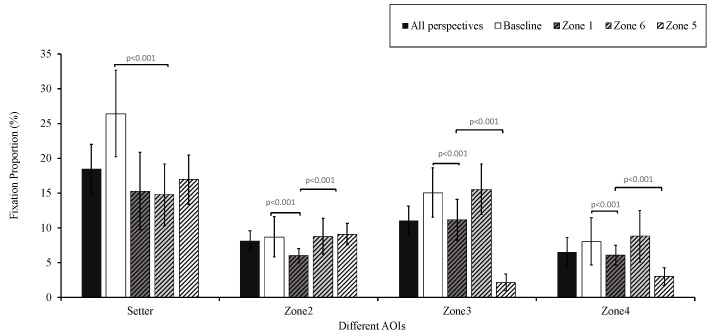
Areas of interest among perspectives (pre-spiking). Note: the *p*-value above the solid line indicates the significance of the difference between viewing perspectives.

A repeated-measures ANOVA was performed with factors 3 (group) × 4 (perspective) × 4 (AOI). The interactions among the three factors (*F*(18,56) = 1.395, *p* = 0.171) and between group and AOIs were not significant (*F*(6,68) = 1.566, *p* = 0.170). However, the main effect of the AOI was significant (*F*(3,35) = 123.108, *p* < 0.001, *η*^2^ = 0.918). The fixation proportion on the setter (18.4% ± 3.6%) was significantly higher than that on other AOIs (Zone 2: 8.1% ± 1.4%; Zone 3: 11.0% ± 2.1%; Zone 4: 6.5% ± 2.1%). The interaction between perspectives and AOIs was significant (*F*(9,29) = 74.962, *p* < 0.001, *η*^2^ = 0.959). Simple effects analysis revealed significant differences among the AOIs from all four perspectives (setter, *F*(3,35) = 41.163, *p* < 0.001, *η*^2^ = 0.779, Zone 2, *F*(3,35) = 36.990, *p* < 0.001, *η*^2^ = 0.760, Zone 3, *F*(3,35) = 174.642, *p* < 0.001, *η*^2^ = 0.937, Zone 4, *F*(3,35) = 81.795, *p* < 0.001, *η*^2^ = 0.875) (Figure 7, Table S5).

#### 3.3.2. Spiking Phase

The AOIs during this phase included the spiker’s trunk, head, shoulder, and wrist–arm, and the ball. A repeated-measures ANOVA conducted for 3 (group) × 4 (perspective) × 5 (AOIs) revealed no significant interaction among the three factors (*F*(24,50) = 0.560, *p* = 0.938). The interaction between group and AOI was also not significant (*F*(8,66) = 0.754, *p* = 0.644).

**Figure 8:**
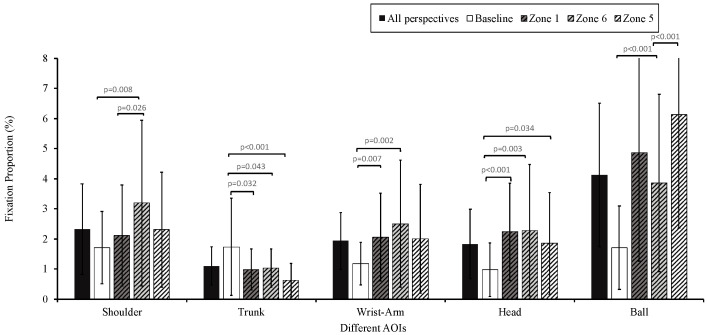
Areas of interest among perspectives (spiking). Note: the *p*-value above the solid line indicates the significance of the difference between viewing perspectives.

The interaction between the AOI and perspective was significant (*F*(12,26) = 11.024, *p* < 0.001, *η*^2^ = 0.836). Simple effects analysis revealed significant differences across perspectives for different AOIs, including the shoulder (*F*(3,35) = 4.011, *p* = 0.015, *η*^2^ = 0.256), trunk (*F*(3,35) = 8.239, *p* < 0.001, *η*^2^ = 0.414), wrist–arm (F(3,35) = 8.472, *p* < 0.001, *η*^2^ = 0.421), head (*F*(3,35) = 11.303, *p* < 0.001, *η*^2^ = 0.492), and ball (*F*(3,35) = 19.407, *p* < 0.001, *η*^2^ = 0.625). The main effect of the AOI was significant (*F*(4,34) = 34.573, *p* < 0.001, *η*^2^ = 0.803). The fixation proportion was highest for the ball (4.1% ± 2.4%) and lowest for the trunk (1.1% ± 0.6%), with similar levels for the shoulder (2.3% ± 1.5%), wrist–arm (1.9% ± 1.0%), and head (1.8% ± 1.2%). The main effect of perspective was also significant (*F*(3,35) = 28.879, *p* < 0.001, *η*^2^ = 0.712), with the baseline perspective (1.5% ± 1.2%) having significantly lower fixation compared to the on-court perspectives (Zone 1: 2.4% ± 2.0%, Zone 6: 2.6% ± 2.3%, Zone 5: 2.6% ± 2.2%) (Figure 8, Table S6).

## 4. Discussion

### 4.1. Superior Response Accuracy but Similar Response Times

The CE and SE groups outperformed the RA of the novice group, indicating that through prolonged training experience, elite experts develop context-specific cognitive search patterns. Experts are more skilled than novices in utilizing early visual cues to anticipate opponents’ actions, a finding that aligns with previous research [7,53,54].

However, the disparity between the CE and SE groups was mainly observed from the on-court perspective, particularly for Zone 1. Furthermore, the RA was lowest from the Zone 1 perspective, indicating the highest difficulty. Since the Zone 4 spiker is generally the main attacker in volleyball tactics, one to two players are often in the front jump to block. The jumping blockers frequently obscure the view of players in Zone 1, which is the closest zone to the main spiker.

Experts can efficiently utilize early cues to anticipate the ball’s trajectory or opponent’s actions more rapidly, leading to shorter RTs than novices [55,56]. However, no significant advantage in RT was found for either CEs or SEs compared to novices in the present study. This may be due to the video pausing when the spiker comes into contact with the ball, which is in the later phase of the spiking process. Most participants, including experts, made their judgment after this moment since they prioritized accuracy over speed. They preferred to watch the entire 2.7 s video before making an accurate decision. Future studies should incorporate additional temporal occlusion points [39], dividing the spiking process into detailed phases to explore whether experts make judgments more quickly with limited information.

### 4.2. Whole Fixation Variables

The present study indicated that the participants in the CE group exhibited fewer fixations, reflecting a stable and efficient visual search strategy among experts. This observation aligns with previous comprehensive studies [2,4]. Moreover, CEs may adjust their visual search patterns (e.g., first fixation duration) based on different perspectives.

From the baseline perspective, the number of fixations was the smallest but the total fixation duration was the longest for all groups. Volleyball players typically focus on a visual pivot and utilize their peripheral vision to gather visual information [34,41]. In this study, baseline videos offered a broader and more stable field of view, and the key tactical information related to spiking, such as setters and spikers, was at the center of the video. Therefore, by focusing their gaze on the central area, players could effectively perceive tactical movements through their peripheral vision and rapidly shift their focus to the key spiking zone as needed [10,14,43]. This could explain why the participants preferred fewer fixations and longer total fixation durations to achieve a more stable visual search from the baseline perspective. Notably, the first fixation duration of the CEs from the baseline perspective significantly exceeded those observed from on-court perspectives. CEs are likely more adept than SEs and novices at utilizing their peripheral vision for global perception; this, combined with foveal vision [57], facilitates a more efficient prediction from the baseline perspective.

The highest number of fixations was observed from the Zone 1 perspective, likely because of its closer proximity to the main spiker, as the proximity to the target influences the number of fixations [58]. Another possible reason could be the high difficulty level and high cognitive load faced by players in Zone 1. These players are responsible for not only defending the straight-line spikes, but also anticipating whether the attacker will perform a feint such as a drop shot [10]. Therefore, they require a more extensive search for visual information, making the anticipatory aspect most challenging. The number of fixations increases correspondingly with task difficulty [13,15]. This insight offers guidance for future training, indicating that coaches should emphasize anticipatory training in Zone 1. In addition, future experimental designs could incorporate the anticipation of attack types, such as a spike or a drop shot, to intricately investigate the cognitive processes from Zone 1.

### 4.3. Areas of Interest from Different Perspectives

Volleyball anticipation research has faced a contentious debate regarding whether experts and novices differ significantly in the visual allocation within AOIs. A study exploring blocker anticipation [39] found that experts focused on the leading spiker earlier and for longer than novices. Another study on setter pass anticipation [38] observed that experts focused more intently on the setter’s hands, whereas novices tended to pay more attention to the ball’s trajectory. This disparity underscored the experts’ enhanced perceptiveness toward task-related kinematic information, as highlighted in a previous study [44]. A study on volleyball anticipation [34] found no significant difference in the fixation proportion on AOIs between expert setters and novices; however, experts demonstrated a more rapid orientation toward key AOIs. A study on setter pass anticipation [41] also found that attention allocation on AOIs was similar across expert, semi-expert, and novice groups. Although the present results did not reveal significant differences between experts and novices either, they did show differences in AOIs from various perspectives, further indicating the necessity of our perspective–specific research.

During the pre-spike phase, the participants exhibited the longest fixation duration on the setter. This difference can be explained by habitual offensive and defensive tactics. The setter, as the decision maker in spiking, determines the direction of the pass and the final spiker [34,36,59], encapsulating substantial critical tactical information. Previous studies [34,39] have delineated the gaze trajectory on AOIs during the pre-spiking phase: ball–setter–ball–spiker. In these studies, the participants initially directed their focus to the ball to evaluate the quality of the pass. Subsequently, they shifted their attention to the setter. By interpreting the setter’s movements, they could predict the direction of the pass and then redirect their gaze to the spiker, anticipating the spike’s trajectory and potential outcomes [41]. These findings are consistent with the results of the present study regarding pre-spiking AOIs. Furthermore, the present study found that from Zone 1, less attention was paid to the Zone 2 spiker. Similarly, from Zone 5, less attention was directed toward the spikers in Zones 3 and 4. This may suggest that defenders were more likely to be drawn to spikers closer to them, potentially overlooking those who were farther away.

During the spiking phase, the participants paid more attention to the ball and the parts of the spiker close to it, such as their shoulders and wrist–arm, but less focus was placed on the spiker’s trunk. Previous studies [14,39] indicated that the spiker’s kinetics and body movements are critical visual cues for predicting the trajectory and endpoint of the spike. Interviews with beach volleyball experts [10] indicated that body orientation, shoulders, arms, elbows, wrists, hands, and the ball are key elements in defensive anticipation. Among these, the ball, hands, and shoulders are mentioned most frequently, underscoring their critical role in predicting defensive plays. In serve receiving [43], expert volleyball players focus more on the server’s shoulder and arm and exhibit a quicker tracking response during the flight phase of the ball. However, some studies [28,60,61,62] have also highlighted that when anticipating the endpoint of the ball, tennis and volleyball experts adopt a strategy of global and peripheral cognition of the attackers’ movements rather than relying on detailed observation. Therefore, given the reduced size and fewer details of spikers in the baseline videos, the participants focused on the trunk as a pivot, attempting to adopt a global perception approach for anticipation. Moreover, the participants focused more on the ball from Zones 1 and 6. A possible reason was that visual information on the spiker’s shoulders and arms was often obscured by jumping blockers, compelling participants to focus more on the ball to anticipate the ball’ trajectory.

### 4.4. Limitations, Strengths, and Future Studies

This study has derived important conclusions, indicating significant differences in accuracy and visual search patterns based on expertise. Moreover, it was found that different perspectives lead to variations in the attention placed on AOIs, underscoring the necessity and importance of studying defense anticipation from various perspectives. With the advancement of technology, virtual reality combined with eye-tracking devices can be used to create more immersive and repeatable scenarios, enriching the empirical research on eye movements in volleyball [63,64]. However, the present study has some limitations. For instance, it did not subdivide the spiking process into more detailed temporal phases. Future research could add more temporal breakpoints to examine gaze behaviors from different perspectives. Additionally, the difficulties in anticipation observed from the on-court perspective, particularly for Zone 1, may be investigated by incorporating more specific tasks for Zone 1, such as distinguishing between a spike or a drop shot, to delve deeper into the participants’ cognitive processes. Intervention studies such as cognitive or perceptual training may also be used to train athletes in predicting volleyball spikes by using different AOI instructions based on different perspectives [65]. During fixations, the human eye undergoes involuntary movements known as micro-saccades, an important metric in cognitive psychology research. These micro-saccades are extremely small eye movements, with amplitudes of generally less than 0.1 degrees in visual angle [66]. As our study focused on training recommendations and guidelines, we did not measure these data. However, they may serve as a potential indicator for future athlete selection. Therefore, measuring this metric in future research to ascertain its validity could prove beneficial.

## 5. Conclusions

The present study indicated that competitive elite and semi-elite players exhibited higher accuracy than novices. Competitive elite players used fewer fixations than both semi-elite players and novices, indicating that their superior performance was related to stable visual search patterns. Across all skill levels, participants showed similar visual allocation among areas of interest (AOIs). However, distinct visual search patterns and differences in AOI allocation were observed between baseline and on-court perspective videos. From the baseline perspective, the participants primarily utilized global perception and peripheral vision, focusing more on the setter zone or the spiker’s trunk. Conversely, they employed more fixations in the on-court perspective, focusing more intensely on the spiker’s detailed movements.

## Figures and Tables

**Table 1:** Description of experimental stimulus videos.

Spiker + Spike Type	Baseline	Zone 1	Zone 6	Zone 5	Total
Zone 2 (RS) + Crosscourt	5	5	5	5	20
Zone 2 (RS) + Midline	2	2	2	2	8
Zone 2 (RS) + Straight	4	4	4	4	16
Zone 3 (MS) + Crosscourt	1	1	1	1	4
Zone 3 (MS) + Midline	4	4	4	4	16
Zone 4 (OS) + Crosscourt	2	2	2	2	8
Zone 4 (OS) + Midline	5	5	5	5	20
Zone 4 (OS) + Straight	3	3	3	3	12
Total	26	26	26	26	104

## Data Availability

The data in this study are available upon request by sending an e-mail to the corresponding author or the first author.

## Supplementary Materials

The following supporting information can be downloaded at: https://www.mdpi.com/article/10.3390/bs14030163/s1, Table S1: Response accuracy (%) rates by group and perspective; Table S2: Total fixation duration (ms) among perspectives; Table S3: Number of fixations by group and perspective; Table S4: First fixation duration (ms) by group and perspective; Table S5: AOIs fixation proportion (%) among perspectives in pre-spiking phase; Table S6. AOIs fixation proportion (%) among perspectives in spiking phase.
