# Supplementary material for: Expert Performance in Action Anticipation: Visual Search Behavior in Volleyball Spiking Defense from Different Viewing Perspectives
Source: Behav Sci (Basel). 2024 Feb 22;14(3):163. doi: 10.3390/bs14030163 (PMC10968438; doi:10.3390/bs14030163)
Supplement: Supplementary file 1 [file behavsci-14-00163-s001.zip › behavsci-2840425-supplementary.pdf]

Supplementary

Table S1. Response accuracy (%) rates by group and perspective.

	Competitive elite		Semi elite		Novices		All participants	
	Mean	SD	Mean	SD	Mean	SD	Mean	SD
All perspectives	58.5	3.25	56.2	5.01	49.9	4.82	55.24	5.60
Baseline	61.90	4.69	61.54	8.08	53.87	7.29	59.45	7.50
Zone 1	59.34	6.35	50.30	5.32	43.36	8.79	51.62	9.36
Zone 6	52.47	9.25	57.10	8.14	48.95	7.71	53.04	8.86
Zone 5	60.16	5.14	55.92	8.25	53.15	12.38	56.68	9.02

Table S2. Total fixation duration (ms) among perspectives.

	All participants	
	Mean	SD
Baseline	2,512.51	357.66
Zone 1	2,358.31	379.35
Zone 6	2,294.81	425.61
Zone 5	2,305.99	434.00

Table S3. Number of fixations by group and perspective.

	Competitive elite		Semi elite		Novices		All participants	
	Mean	SD	Mean	SD	Mean	SD	Mean	SD
All perspectives	7.42	1.34	8.25	1.06	9.22	0.93	8.22	1.45
Baseline	6.83	1.23	7.61	1.16	8.86	1.20	7.68	1.43
Zone 1	7.98	1.36	8.88	1.27	9.63	1.12	8.76	1.41
Zone 6	7.45	1.73	8.25	1.00	9.26	1.05	8.25	1.49
Zone 5	7.43	1.48	8.28	1.19	9.16	1.31	8.22	1.48

Table S4. First fixation duration (ms) by group and perspective.

	Competitive elite		Semi elite		Novices	
	Mean	SD	Mean	SD	Mean	SD
All perspectives	264.76	84.92	256.59	44.02	231.30	47.82
Baseline	296.52	72.35	283.24	87.64	237.86	56.94
Zone 1	221.42	52.16	260.60	76.07	212.36	47.16
Zone 6	242.23	53.00	268.01	104.67	237.38	54.55
Zone 5	239.00	55.05	276.63	91.11	237.34	48.74

Table S5. AOIs fixation proportion (%) among perspectives in pre-spiking phase

Perspective / AOIs	All		Baseline		Zone1		Zone 6		Zone 5	
	Mean	SD	Mean	SD	Mean	SD	Mean	SD	Mean	SD
Setter	18.43	3.62	26.44	6.20	15.28	5.56	14.76	4.47	16.94	3.53
Zone2	8.12	1.42	8.68	2.88	5.97	1.00	8.75	2.60	9.08	1.54
Zone3	11.02	2.09	15.07	3.57	11.15	2.95	15.54	3.63	2.15	1.20
Zone4	6.49	2.11	8.02	3.39	6.06	1.39	8.78	3.72	3.04	1.26
Shoulder	2.32	1.50	1.70	1.20	2.11	1.68	3.19	2.76	2.31	1.91
All1	11.01	2.45	14.55	4.21	9.61	3.26	11.96	3.67	7.80	2.11

Notes: AOIs, areas of interest.

Table S6. AOIs fixation proportion (%) among perspectives in spiking phase

Perspective / AOIs	All		Baseline		Zone1		Zone 6		Zone 5	
	Mean	SD	Mean	SD	Mean	SD	Mean	SD	Mean	SD
Shoulder	2.32	1.50	1.70	1.20	2.11	1.68	3.19	2.76	2.31	1.91
Trunk	1.10	0.63	1.73	1.61	0.97	0.68	1.02	0.64	0.63	0.56
Wrist-Arm	1.93	0.95	1.18	0.71	2.05	1.46	2.50	2.11	2.00	1.81
Head	1.83	1.16	0.98	0.89	2.23	1.62	2.28	2.19	1.85	1.69
Ball	4.12	2.38	1.71	1.39	4.86	3.60	3.85	2.95	6.14	3.78
All AOIs	2.26	1.45	1.46	1.20	2.44	2.05	2.57	2.28	2.58	2.21

Notes: AOIs, areas of interest.
